# Controlled vs Spontaneous Ventilation for Bronchoscopy in Children with Tracheobronchial Foreign Body

**Published:** 2017-11

**Authors:** Leila Mashhadi, Alireza Sabzevari, Mohammad Gharavi Fard, Reza Shojaeian, Maryam Salehi, Marjan Joodi, Mahdi Fathi, Ali Jafarzadeh Esfehani, Kamran Khazaeni

**Affiliations:** 1 *Department of Anaesthesiology, Mashhad University of Medical Sciences, Mashhad, Iran.*; 2 *Department of Paediatric Surgery, Sarvar Children’s Hospital, Mashhad University of Medical Sciences, Mashhad, Iran.*; 3 *Research Center for Patient Safety, Mashhad University of Medical Sciences, Mashhad, Iran.* * And Clinical Research Unit, Ghaem hospital, Faculty of Medicine, Mashhad University of Medical Sciences. *; 4 *Medical School and Neonatal Research Center, NICU of Emamreza Hospital, Mashhad University of Medical Science, Iran.*; 5 *Department of Otolaryngology, Head and Neck Surgery, Mashhad University of Medical Sciences, Mashhad, Iran.*

**Keywords:** Children, Controlled ventilation, Respiratory aspiration, Spontaneous ventilation

## Abstract

**Introduction:**

Tracheobronchial foreign body aspiration is a common life-threatening condition in children. There are controversies in the management of this condition, including the type of ventilation during bronchoscopy. This study aims to compare anesthesia with controlled ventilation versus spontaneous ventilation in rigid bronchoscopy in children with foreign body aspiration.

**Materials and Methods:**

Patients who were candidates for rigid bronchoscopy due to foreign body aspiration were randomly assigned to either anesthesia with spontaneous ventilation or controlled ventilation. End tidal CO_2_ (ETCO_2_), electrocardiogram (ECG), heart rate (HR), oxygen saturation (SpO_2_), non-invasive blood pressure (NIBP) and complications and accidents during the surgery and recovery were recorded for each patient. Surgeon comfort during the procedure was also evaluated for each patient. A 20% change in HR or NIBP was considered significant. SpO_2_ values under 90% are considered desaturation.

**Results:**

Fifty-one patients (31 male and 20 female) entered the study. The mean age was 26.76 months, ranging from 6 to 100 months. Choking and cough were present in 94% and 96.1% of the patients, respectively. Nuts were the most common foreign body (76.9%). The controlled ventilation group had significantly fewer complications, and surgeon comfort was significantly higher in this group. Oxygen desaturation was significantly more prevalent in the spontaneous ventilation group during laryngoscopy and bronchoscopy (P<0.001).

**Conclusion:**

Controlled ventilation has the potential to be used as an effective alternative option in anesthesia for patients with suspected foreign body aspiration.

## Introduction

Tracheobronchial foreign body aspiration is a relatively common medical problem in children. Otolaryngologists, pediatric surgeons and anesthesiologists are involved in the management of these children (1-3). The incidence of tracheobronchial foreign body aspiration was reported to be 1,908 ± 273 cases per year during 2009 and 2011 in the United States (2). Nearly 52% of children with tracheobronchial foreign body aspiration are between the age of 1 and 3 years (1,2). Regardless of recent improvements in diagnostic procedures as well as anesthetic and bronchoscopy techniques, the morbidity and mortality of foreign body aspiration is still considerable, mainly due to the nature of the condition and the choice of treatment options (4). The morbidity and mortality of tracheobronchial foreign body aspiration is estimated to be 10% (1). Tracheobronchial foreign body aspiration problems are aggravated by delayed or wrong clinical diagnosis, variations in types of foreign bodies and wrong clinical management of foreign body aspiration (3,4).

Major complications of tracheobronchial foreign body aspiration are inevitable regardless of prompt diagnosis and management; therefore the focus of attention should be on preventing aspiration (4). On the other hand, some of the complications of tracheobronchial foreign body aspiration are potentially preventable by the appropriate choice of diagnostic and management techniques (4,5). 

Major controversies exist in terms of the different levels of management of foreign body aspiration in children, including the emergent or urgent management approach to foreign body aspiration, choice of diagnostic procedures before bronchoscopy, type of bronchoscopy (rigid vs flexible bronchoscopy) and type of induction and maintenance of anesthesia (4). As stated earlier, there is no general agreement on the choice of anesthetic procedure in the management of foreign body aspiration in children. The findings of previous studies that assessed the efficacy of two major types of anesthesia, including spontaneous ventilation and controlled ventilation, have led to divergent conclusions. A number of previous studies found no difference in the efficacy and complications between spontaneous ventilation and controlled ventilation in the management of foreign body aspiration (6–8).These findings have led to the recommendations that the choice of the technique for anesthesia should be based on the expertise of the anesthesiologist (4,9,10). In contrast to these observations, a more recent meta-analysis identified a significantly higher frequency of laryngospasm in the spontaneous ventilation compared with controlled ventilation (11). This controversy in the findings of previous studies was mainly due to the small sample size of the studies, and a lack of proper randomization and controlling for confounding factors. The aim of this study was to compare the advantages and disadvantages of spontaneous ventilation with controlled ventilation in tracheobronchial foreign body aspiration in children.

## Materials and Methods

**Fig 1:**
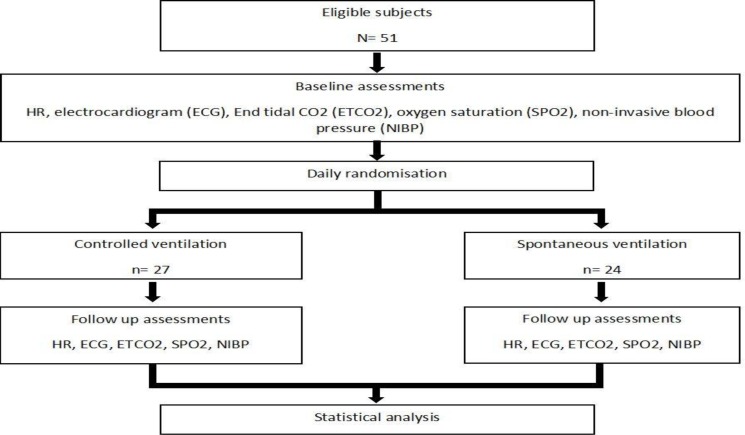
Flowchart of the study

*Study population: *This randomized clinical trial was carried out in children who were candidates for rigid bronchoscopy in Sheikh Hospital, Mashhad, Iran during 2015–2017. Subjects were randomly assigned to controlled ventilation or spontaneous ventilation groups. Subjects were approached by a researcher, and the procedure of anesthesia was described for the parents. Parents who were willing to participate in the study signed an informed consent prior to participation. The study procedure was approved by the Mashhad University of Medical Sciences Ethical Committee (IR.MUMS.REC.1394.88). All children with documented foreign body aspiration who were candidates for rigid bronchoscopy were included in the study. Subjects with a foreign body located in the laryngeal aditus or hypopharynx were excluded due to the short duration of anesthesia. A flowchart of the study is shown in Figure 1.

Based on the observed type I and II errors (5 and 20 respectively) in a study by Soodan et al. (8), the sample size was calculated according to the appropriate sample size formula (12). The sample size was calculated as 25 subjects in each study group and, considering 20% drop-out, the final sample size was calculated 25×1.2=30 subjects in each group. Randomization was performed daily based on the number of admitted children meeting the inclusion criteria until reaching the minimum sample size due to the time restrictions. Sampling was continued in case any subject dropped out or was excluded from the study.

*Study design*

Intervention and control groups were both pre-oxygenated prior to anesthesia, using 100% oxygen.

The controlled ventilation group received 3mg/kg sodium thiopental and 1µg/kg fentanyl while being ventilated. Atracurium was administered at a dose of 0.5 mg/kg in order to induce skeletal muscle relaxation in case there was no problem in ventilation. Subjects then underwent rigid bronchoscopy. Maintenance of anesthesia was achieved with propofol at 80–100 µg/kg/min. In this procedure, oxygen was administered at 5–10 l/min via the T-segment of the bronchoscope. The depth of anesthesia was adjusted based on hemodynamic changes, including heart rate (HR), respiratory rate (RR), lacrimation, perspiration, and movements of subjects as well as cough and bucking. In case of prolonged hypoxia (oxygen saturation below 90% for 2 minutes) or inability to maintain required depth of anesthesia that made bronchoscopy problematic, the procedure was interrupted and subjects were ventilated until returning to the normal condition.

In the spontaneous ventilation group, anesthesia was induced using a face mask with 8% sevoflurane and 1µg/kg fentanyl intravenously. Then, sevoflurane concentration was reduced to 3%. When an acceptable depth of anesthesia was reached, rigid bronchoscopy was initiated. Afterwards, 100% oxygen and 3% sevoflurane were administered via the T-part of the bronchoscope.

Subjects were intubated after the bronchoscopy was terminated and were awakened by administration of neostigmine and atropine. Subjects were transferred to the recovery unit only if the Steward’s recovery score was ≥5. HR, RR and SPO_2_, as well as cough episodes, were recorded for 1 hour during the subjects stay in the recovery unit. Complications of the procedures including bucking, laryngospasm, laryngeal edema, cough, bronchospasm, seizures and pneumothorax as well as breathing arrest, cardiac arrhythmias, pneumomediastinum, body movements, airway reflexes and foreign body replacement were also recorded during the stay in the recovery unit. Both groups underwent continuous monitoring for HR, electrocardiogram (ECG), end tidal CO_2_ (ETCO_2_), oxygen saturation (SPO_2_) while non-invasive blood pressure (NIBP) was monitored every 5 minutes. A 20% change in HR, systolic or diastolic blood pressure was considered a significant change. Arterial desaturation was defined as SPO_2 _<90%. Any significant event or complication during and at the end of surgery was recorded for each subject. Surgeons were blinded with respect to the type of anesthesia and ventilation prior to the initiation of surgery. They did not have any influence in the choice of anesthesia. They were also asked to report whether they were satisfied with the rigid bronchoscopy procedure at the end of surgery for subjects in both groups. Therefore, surgeon satisfaction variable was recorded as a binary categorical variable.

*Statistical analysis*

Data were analyzed using the statistical package for social sciences (SPSS) version 21 (IBM, Inc, Chicago, IL, USA). Continuous data were assessed for normality using the Shapiro-Wilk test. Continuous data were presented as mean and standard deviation (SD) in case of normality, and the median and interquartile range (IQR) were used to describe non-normally distributed continuous data. Categorical variables were described as frequency and percentage. The independent t-test was used to compare normally distributed continuous variables between study groups, while the Mann-Whitney test was used to compare the non-normally distributed variables between groups. The chi-square and Fisher exact tests were used to assess the difference in distribution patterns of categorical variables in the study groups. Values of p less than 0.05 were considered statistically significant.

## Results

Among the subjects, 27 (52.9%) were assigned to the controlled ventilation group and 24 (47.1%) were assigned to the spontaneous ventilation group. Baseline characteristics of the study subjects and a comparison between study groups are shown in Table 1.

**Table 1:** Characteristics of subjects at baseline and their comparison between study groups

Variable		Controlled ventilation n=27	Spontaneous ventilation n=24	P value
Age (months)†		27.00 (27.00)	28.00 (23.00)	0.22
Gender	Male	9(33.3%)	15(62.5%)	0.81
	Female	18(66.7%)	9(37.5%)	
Weight (kg)		11.59±5.4	11.22±2.5	0.70
Foreign body diameter (mm)		6.91±2.8	7.356.91±3.6	0.68

kg= kilogram, mm= millimetre

† Median and interquartile range (IQR) were described and the Mann-Witney test was used for comparison

A total of 51 subjects (31 [60.8%] males and 20 [39.2%] females) were recruited in the study. There was no significant difference in terms of gender distribution between the intervention and control groups (15 males and 12 females in the intervention group vs nine males and 15 females in the control group, P=0.81) (Table 1).

**Table 2:** Frequency and percentage of signs and symptoms of foreign body aspiration and their comparison among study groups

**Variable**	**Controlled ventilation** **n=27**	**Spontaneous ventilation** **n=24**	**P value**
Cough	25(92.6%)	22(95.7%)	0.65
Fever	26(96.3%)	23(95.8%)	0.93
Hoarseness	8(29.6%)	15(62.5%)	0.02*
Tachypnea	7(25.9%)	7(29.2%)	0.80
Wheezing	8(29.6%)	14(58.3%)	0.04 *
Intercostal retraction	1(3.7%)	1(4.2%)	0.93†
Respiratory distress	19(70.4%)	20(83.3%)	0.28
Cyanosis	9(33.3%)	9(37.5%)	0.76
Stridor	6(22.2%)	6 (25.00%)	0.81

* Significant difference in distribution pattern of the variable between groups based on the chi-square test

† Fisher exact test was used for the analysis

The patients were 6–100 months old, with a mean age of 26.76 ± 12.57 months. The mean age was 27 months in the intervention group, compared with 23 months in the control group. The observed difference was not statistically significant (P=0.22).

The observed signs and symptoms of subjects and their comparison between study groups are presented in Table 2. There was no significant difference in the distribution of signs and symptoms between the intervention and control groups, except for in hoarseness and wheezing which were significantly more prevalent in the control group (P=0.02 and P=0.04, respectively) (Table 2). A history of choking was reported in 47 (92.2%) of subjects.

**Fig 2:**
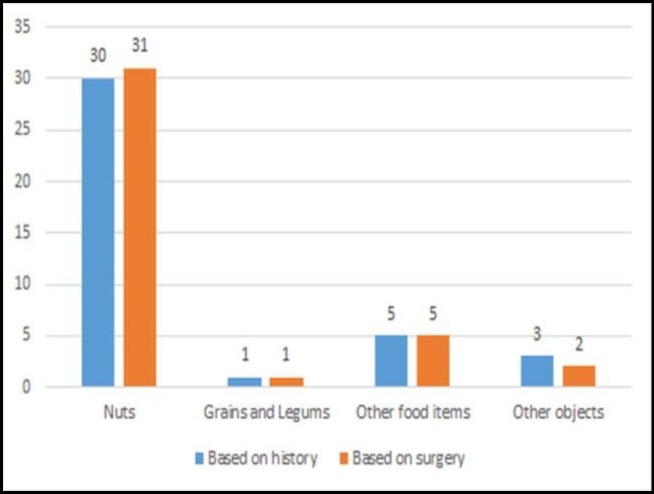
Frequency of foreign body types as per study groups

Types of aspirated foreign bodies are presented in Figure 2. Nuts, as the most common foreign bodies aspirated, were compared between the two groups, and no statistically significant difference was found.

The foreign body was located on the right side in 33.3% of subjects, while 30.7% of subjects had the foreign body aspirated on the left side. In 24 (68.6%) subjects, the obstruction was below 50% of the lumen while in nine (25.7%) subjects, obstruction was between 50% and 70% and in two (5.7%) subjects the obstruction was more than 70%.

A total of 25 (92.6%) subjects in the intervention group had no complications, while only one subject in the control group had no complication. There was a significant association between having a complication and being in the control group (i.e. spontaneous ventilation) (P<0.001). Complications during bronchoscopy and their comparison between study groups are presented in Table 3.

 Changes in study parameters at the commencement of laryngoscopy and rigid bronchoscopy and at the end of rigid bronchoscopy are presented in Table 4. Changes in SPO_2_ was significantly more frequent in the control group (i.e. spontaneous ventilation) at the end of rigid bronchoscopy (P<0.001) (Table.4).

Surgeons were significantly more satisfied in the controlled ventilation (intervention) group. (27 [100.0%] satisfaction in the intervention group against 3 [12.5%] satisfaction in the control group, P<0.001).

**Table 3:** Frequency of complications during rigid bronchoscopy and their comparison between study groups

**Variable**	**Controlled ventilation** **n=27**	**Spontaneous ventilation** **n=24**	**P value**
Bucking	0 (0.0%)	22(91.7%)	<0.001*
Laryngospasm	0(0.0%)	17(70.8%)	<0.001*
Laryngeal oedema	0(0.0%)	9(37.5%)	<0.001*
Cough	0(0.0%)	0(0.0%)	-
Bronchospasm	0(0.0%)	3(12.5%)	0.06
Seizure	0(0.0%)	0(0.0%)	-
Strain	0(0.0%)	0(0.0%)	-
Pneumothorax	0(0.0%)	0(0.0%)	-
Arrhythmia	0(0.0%)	4(16.7%)	0.03 *
Breathing arrest	0(0.0%)	1(4.2%)	0.28
Pneumomediastinum	0(0.0%)	0(0.0%)	-
Body movement	0(0.0%)	1(4.2%)	0.28
Airway reflexes	0(0.0%)	17(70.8%)	<0.001 *
Foreign body replacement	2(7.4%)	11(45.8%)	0.002 *

* Significant difference in distribution pattern of the variable between groups based on the Fisher exact test

**Table 4:** Frequency of complications at the commencement of laryngoscopy and rigid bronchoscopy and at the end of rigid bronchoscopy and their comparison between study groups

**Variable**	**Controlled ventilation** **n=27**	**Spontaneous ventilation** **n=24**	**p value**
Significant HR change at the beginning of laryngoscopy	6(22.2%)	1(3.0%)	0.36
Significant HR change at the beginning of bronchoscopy	5(18.5%)	10(41.7%)	0.07
Significant HR change at the end of bronchoscopy	2(7.4%)	2(8.7%)	0.87
Significant SBP change at the beginning of laryngoscopy	0(0.0%)	0(0.0%)	-
Significant SBP change at the beginning of bronchoscopy	0(0.0%)	0(0.0%)	-
Significant SBP change at the end of bronchoscopy	0(0.0%)	0(0.0%)	-
Significant SPO_2_ change at the beginning of laryngoscopy	1(3.7%)	1(4.2%)	0.93
Significant SPO_2_change at the beginning of bronchoscopy	0(0.0%)	3(12.5%)	0.06
Significant SPO_2_change at the end of bronchoscopy	0(0.0%)	9(37.5%)	P<0.001*
Significant changes in SPO_2 _in recovery	1(3.7%)	0(0.0%)	0.35

HR= Heart Rate, SBP= Systolic Blood Pressure, SPO_2_= Oxygen Saturation

* Significant difference in distribution pattern of the variable between groups based on the Fisher exact test

## Discussion

Considering the prevalence of tracheobronchial foreign body aspiration and its high morbidity and mortality, the appropriate prevention, diagnosis and treatment of this condition may have a significant role in the physical, psychological and social aspects of families (13,14). 

There is no general agreement on the choice of safe anesthesia technique in tracheobronchial foreign body aspiration (4,5). Traditionally, anesthesiologists have maintained spontaneous ventilation in the patients. This is based on the hypothesis that spontaneous ventilation might help anesthesiologists in the case of ventilation problems and reduce the risk of later complications by reducing the vital time for the management of the condition (4,11). This idea is not universally supported because spontaneous breathing may cause problems, among which increased risk of reflexes during laryngoscopy and bronchoscopy and involuntary movement of the patient due to insufficient depth of anesthesia are the most important (4,15). In contrast, these conditions are theoretically overcome by the use of controlled ventilation, but this technique poses a new threat to the surgeon, which is lack of spontaneous breathing in the case of airway loss and also the possibility of distal displacement of a non-obstructing foreign body in the bronchial tree.

In this randomized controlled clinical trial, subjects were randomized in terms of equal confounding variables (including age, gender, weight, type and diameter of the foreign body as well as delay before the initiation of the treatment) as demonstrated by the statistical similarity between groups in terms of these variables (P>0.05). This study design provides the opportunity to perform simple statistical analyses with a high precision on a small sample size, as there was no need for including more subjects in order to perform sophisticated statistical analyses with statistical adjustments.

This study revealed that the most common aspirated foreign body was nuts followed by grains and legumes and other food items. This finding was in line with the findings of a previous study that identified vegetable matters as the most common type of foreign body (16). This study also found a slightly higher frequency of foreign bodies on the right side, which is also in line with the findings of a previous study (16).

This study revealed that the overall prevalence of complications was significantly lower in the controlled ventilation group compared with the spontaneous ventilation group. In particular, the frequency of bucking, airway reflexes, laryngospasm, laryngeal edema, foreign body replacement and arrhythmia were significantly lower in the controlled ventilation group compared with the spontaneous ventilation group. These findings were in line with the findings of a previous meta-analysis that identified a significantly higher frequency of laryngospasm with spontaneous ventilation compared with controlled ventilation (11). Unlike the findings of this study, previous studies have found no difference between spontaneous ventilation and controlled ventilation in the management of foreign body aspiration (4,9,10). In one study it was reported that controlled ventilation was not superior to spontaneous ventilation, and that the choice between these procedures depends on the preference of the anesthesiologist (4).

This study revealed no significant difference in hemodynamic status between the groups, except for SPO_2 _at initiation of rigid bronchoscopy. Previous studies have reported controversial findings in terms of hemodynamic effects of controlled and spontaneous ventilation. In contrast to the findings of this study, a recent meta-analysis reported no significant difference in oxygen desaturation between spontaneous and controlled ventilation (11). Furthermore, in another study on 83 patients with tracheobronchial foreign body aspiration, respiratory complications were present in 18% of subjects who underwent total intravenous anesthesia and assisted ventilation (17). These complications included reduced oxygen saturation, bronchospasm and recurrent pneumothorax (17). Conversely, Soodan et al. (2004) reported that all their subjects in the spontaneous ventilation group (n=36) required assisted ventilation due to reduced oxygen saturation or unacceptable depth of anesthesia (8). In another study in 46 patients with foreign body aspiration who underwent rigid bronchoscopy, spontaneous ventilation was considered safe due to the observed similarities in outcomes of intravenous and inhalation rout of anesthesia (18). They also suggested that prior experience and close cooperation between the otolaryngologist and anesthesiologist should be taken into consideration in the choice of spontaneous or controlled ventilation in the management of foreign body aspiration (18). This study also revealed a significant association between surgeon satisfaction and the controlled ventilation procedure.

Due to the paucity of data on the effectiveness of spontaneous ventilation against controlled ventilation, it is still early to obtain a concise conclusion. However, it can be inferred from the current study that controlled ventilation might be a good and acceptable choice in the management of tracheobronchial foreign body aspiration in children. Furthermore, because surgeon satisfaction was significantly higher with controlled ventilation, this procedure might prove a better approach for surgeons in the bronchoscopic removal of foreign bodies. There is a need for further studies with larger sample sizes in order to assure the safety or possible superiority of controlled ventilation in comparison with spontaneous ventilation.

One of the limitations of this study is the small sample size. Although the sample size of the study reached the minimum required sample size, time restrictions and the emergency nature of the condition prevented the researchers from including more subjects in the study. This limitation may have prevented us from generalizing the results to the whole population of children with foreign body aspiration. Another limitation of the study is the use of a binary scale for surgeon satisfaction due to their busy schedule. Since surgeons have an important role in the management of these emergencies, it is recommended that further studies perform an in-depth assessment of the satisfaction of the surgeons.

## Conclusion

Based on the findings of this study and the currently available data in the literature, it can be deduced that controlled ventilation is a safe procedure in the management of foreign body aspiration in children, and it can be considered an alternative choice for conventional spontaneous ventilation. However, there is a need for further studies to generate a definitive recommendation on the choice of controlled ventilation or spontaneous ventilation in the management of foreign body aspiration in children.
